# Association of periodontitis with handgrip strength and skeletal muscle mass in middle-aged US adults from NHANES 2013–2014

**DOI:** 10.1007/s40520-023-02471-2

**Published:** 2023-06-30

**Authors:** Kübra Kaymaz, Christian Wiessner, Gülistan Bahat, Tugba Erdogan, Alfonso J. Cruz-Jentoft, Antonia Zapf

**Affiliations:** 1 The Center for Experimental Medicine, Medical Biometry and Epidemiology, University Medical Center Hamburg-Eppendorf, Christoph-Probst-Weg 1, 4th Floor, 20246 Hamburg, Germany; 2 Department of Internal Medicine, Istanbul Medical Faculty, Division of Geriatrics, Istanbul University, Istanbul, Turkey; 3 Servicio de Geriatría, Hospital Universitario Ramón y Cajal (IRYCIS), Madrid, Spain

**Keywords:** Periodontitis, Sarcopenia, Frailty, Skeletal muscle mass, Handgrip strength, Epidemiology

## Abstract

**Objectives:**

The relationship between periodontitis and sarcopenia parameters in middle-aged adults is largely unexplored. This study investigated the association between periodontitis and combined handgrip strength and skeletal muscle mass in middle-aged adults.

**Materials and methods:**

A sub-cohort of 1912 individuals with complete periodontal and whole-body dual X-ray absorptiometry examinations from the 2013–2014 wave of the National Health and Nutrition Examination Survey (*n* = 10,175) were analyzed using fully adjusted multiple linear regression models for associations between periodontitis and skeletal muscle mass index (kg/m^2^) and combined handgrip strength (kg).

**Results:**

The mean age of the study cohort was 43 (± 8.4) years and 49.4% of the participants were male. In total, 612 participants (32%) were determined to have periodontitis, of which 513 (26.8%) had non-severe (mild or moderate) periodontitis, and 99 (5.2%) had severe periodontitis. In unadjusted regression models, both non-severe and severe periodontitis were associated with SMMI (*β*_non-severe_ = 1.01, 95% CI 0.50; 1.52 and *β*_severe_ = 1.42, 95% CI 0.59; 2.25) but not with cHGS. After adjusting for age, sex, education, body mass index, bone mineral density, diabetic status, education, total energy intake, total protein intake, and serum vitamin D2 + D3, periodontitis was associated with cHGS (*β*_non-severe_ = -2.81, 95% CI − 4.7; − 1.15 and *β*_severe_ = − 2.73, 95% CI − 6.31; 0.83). The association between periodontitis and SMMI remained for non-severe periodontitis (*β*_non-severe_ = 0.07, 95% CI − 0.26; 0.40 and *β*_severe_ = 0.22, 95% CI − 0.34; 0.78).

**Conclusion:**

The present study highlights the need of further prospective research to investigate the nature and direction of the relationship between periodontitis and sarcopenia indicators**.** Future studies can support the screening, prevention and clinical management of sarcopenia and periodontitis, and emphasize the interdisciplinary and complementary approach between the disciplines of geriatric medicine and periodontology.

**Supplementary Information:**

The online version contains supplementary material available at 10.1007/s40520-023-02471-2.

## Introduction

Periodontitis is a chronic inflammatory disease of the tooth-supporting tissues caused by an oral dysbiosis that, if left untreated, leads to tissue breakdown and tooth loss. It is a major cause of tooth loss in adults and is associated with reduced oral health-related quality of life [1]. Periodontitis has been shown to be an independent risk factor for various non-communicable diseases, such as cardiovascular diseases [2], diabetes mellitus [3], hypertension [4], and chronic kidney disease [5]. Periodontitis has been linked to the manifestation or exacerbation of these conditions through a variety of mechanisms, including increased low-grade systemic chronic inflammation due to periodontal inflammation and infiltration of periodontal pathogens into the circulatory system, resulting in *alio loco* tissue reactions [6].

Sarcopenia is a muscle disorder associated with frailty, decreased mobility, and increased risk of mortality due to loss of muscle mass and function in adults [7–9]. Metabolic complications and the risk of fall injuries increase in sarcopenia patients, causing relevant disability, malnutrition, reduced health-related quality of life, and even mortality [7]. Systemic diseases that increase chronic low-grade systemic inflammation, such as diabetes [8], obesity [10], and metabolic syndrome [11], were associated with sarcopenia, reducing the overall health-related quality of life. A reduction of muscle mass and strength in sarcopenia patients is widely associated with a reduction in anabolic metabolic function, a decline in immune system functions, and increased inflammation [12].

Chronic and inflammation-based diseases and conditions that considerably reduce the quality of life are associated with common risk factors, such as smoking, obesity, a sedentary lifestyle, and low socioeconomic status [13]. Since both periodontitis and sarcopenia are chronic diseases that are associated with common risk factors, we hypothesized that the two conditions could be interrelated. In recent years, an increasing number of studies have reported clear associations between sarcopenia indicators, such as handgrip strength, masticatory functions, and muscle mass, and reduced oral health and oral hypofunction [14]. The number of remaining teeth and higher masticatory function showed negative associations with handgrip strength in older adults in Japan [15]. Edentulism and poor oral health also showed relevant associations with frailty in older adults in Mexico [16]. In addition, tooth loss and masticatory problems were suggested to contribute to a poorer diet and nutritional status, resulting in frailty and sarcopenia [17]. In this context, difficulty eating, having fewer teeth, and edentulism showed considerable associations with frailty in older British men in a nationally representative study [18]. Handgrip strength was recently associated with periodontitis in 30-year-old or older Korean adults [19]. However, the majority of studies relating to the associations between periodontitis and sarcopenia indicators were conducted on older adults aged over 65. There remains a clear gap in the literature regarding associations of sarcopenia indicators and periodontitis in younger adults and in different populations.

In the present study, we will investigate the associations between periodontitis and with handgrip strength or skeletal muscle mass index in a subset of a representatively sampled U.S. population.

## Materials and methods

### Study population

**Fig. 1:**
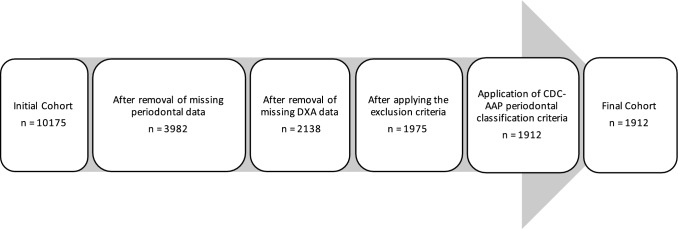
Data selection flowchart diagram

A cross-sectional study was conducted on the adult participants of the 2013–2014 National Health and Nutrition Examination Survey (NHANES). NHANES examines the health and nutritional status of a representative group of children and adults in the USA by conducting household interviews, laboratory and physical examinations. The NHANES datasets are publicly available, and the participants have consented to the use of their data for research purposes. The design and methods of NHANES 2013–2014 have been described elsewhere [20]. The 2013–2014 wave of NHANES was selected because it included both complete periodontal examinations and whole-body dual-energy X-ray absorptiometry (DXA) examinations. Both examinations were available for the cohort that were aged between 30 and 59 years. The reasons for excluding participants from the DXA examination included pregnancy, a history of barium use in the previous 7 days, weight above 204 kg (450 lbs), and height above 198 cm (6′ 5″) [21]. The exclusion criteria for periodontal examination were having less than one natural tooth and any requirement for antibiotic prophylaxis prior to periodontal probing [22]. A more detailed description of the NHANES 2013–2014 cohort can be found in the study published by Eke et al. [23]. In the present study, adults with certain medical conditions were excluded due to their strong association with sarcopenia. The exclusion criteria of the present study included the following: (1) adults with cancer or malignancies (self-reported, *n* = 91) [24], (2) chronic kidney disease (estimated glomerular filtration rate < 30 mL/min, *n* = 0) [25], (3) heart disease (self-reported heart failure, coronary heart disease, coronary heart attack, angina pectoris, ischemic heart disease, and stroke, *n* = 163) [26], and (4) those who were undergoing treatment with systemic corticosteroids (self-reported, *n* = 0) [27]. Data flowchart is presented in Fig. 1.

### Periodontal examination and classification

Gingival recessions (mm) from the cemento-enamel junction and probing pocket depths (PPD, mm) were recorded by licensed dentists at six sites (disto-facial, mid-facial, mesio-facial, disto-oral, oral, and mesio-oral) for each permanent tooth, excluding third molars according to the NHANES protocol [28]. Clinical attachment loss (CAL, mm) was calculated based on the sum of gingival recession and probing pocket depth at each examination site for each tooth, and gingiva height was recorded as a negative value, 0, or a positive value, depending on the position of the gingival margin relative to the CEJ [28]. Periodontitis was classified according to the updated Centers for Disease Control and Prevention/American Academy of Periodontology (CDC/AAP) criteria for periodontitis case definitions [29]. Inter-examiner and intra-examiner calibration data for periodontal parameters in NHANES III have previously been published by Dye et al. [30]. All participants that met the CDC-AAP criteria were classified as “no periodontitis”, “non-severe periodontitis (mild or moderate)” or “severe periodontitis” (*n* = 1912).

### Determination of skeletal muscle mass

The whole-body dual X-ray absorptiometry scans were acquired by trained and certified radiology technicians at the NHANES center using the Hologic Discovery model A densitometer (Hologic, Inc., Bedford, Massachusetts) and assessed using Apex 3.2 software according to the published NHANES protocol [31]. Data on total lean mass without bone mineral content were available in the database and were divided by the value of height squared to allow comparisons between individuals. This calculation was included in the analysis as the skeletal muscle mass index (SMMI). Low SMMI was defined as below 5.5 kg/m^2^ for women and below 7.0 kg/m^2^ for men according to the previously published case definitions for sarcopenia [8].

### Determination of muscle strength

The muscle strength was measured by way of an isometric handgrip test using a dynamometer (Takei Digital Grip Strength Dynamometer, Model T.K.K.5401) in a standing position and involved exhaling while squeezing the dynamometer to avoid increasing intra-thoracic pressure [20]. The test was repeated three times on each hand. The combined handgrip strength (cHGS, kg) was the sum of the largest reading from each hand. Further details about the protocol can be found on the NHANES website [20]. Low cHGS was defined as 16 kg and/or below for women and 27 kg and/or below for men in accordance with the previously published case definitions of sarcopenia [8].

### Covariates

Potential associations with skeletal muscle mass, handgrip strength, and/or periodontitis were considered when selecting covariates. Age (continuous), sex assigned at birth (female, male), total daily energy intake (continuous, kcal/day), daily protein intake (continuous, g/day), and education level (low: 12th grade with no diploma and/or below; medium: high school graduate and/or equivalent and/or some level of higher education; or high: completed college degree and/or above) were self-reported by the participants (for details see https://www.cdc.gov/nchs/NHANES/). Smoking status could not be included in the analysis due to more than 60% missing data of the variable. Body measurements were recorded by trained health technicians, and the body mass index (BMI, kg/m^2^) was calculated based on the weight and standing height of the participants recorded at the NHANES examination center. From laboratory HbA1c results, the diabetes mellitus type II status was included as a categorical variable [HbA1c (%)], non-diabetes: < 5.7; prediabetes: 5.7–6.5; diabetes: > 6.5]. Body mass index (BMI) was categorized according to the World Health Organization (WHO) obesity classification for participant characteristics but was included as a continuous variable in the regression analysis. Bone mineral density (BMD, g/cm^2^) and total serum vitamin D2 and D3 (nmol/L) were reported in the NHANES dataset and were included as continuous variables. An estimated glomerular filtration rate (eGFR) was calculated from standardized serum creatinine using a previously validated equation for the study population [32]. Chronic kidney disease was classified according to the National Kidney Foundation Kidney Disease Outcomes Quality Initiative eGFR reference values [33].

### Statistical analyses

All continuous variables are reported as mean with standard deviation, while categorical variables are reported as values and percentages. All analyses were predicated on investigating the association between periodontitis and its severity as independent variables and SMMI or cHGS as dependent variables using multiple linear regression models. Unadjusted and adjusted associations were reported as regression coefficients with corresponding two-sided 95% confidence intervals (95% CI). Adjusted associations were estimated while accounting for confounding variables that were identified a priori based on biological plausibility and evidence. Interaction terms were included in the models to account for potential effect modifications. In the analyses, a stepwise approach was avoided, and all covariates that were identified a priori were included. In all regression analyses, the complex survey design of NHANES was considered and survey weights, primary sampling units, and strata were included in the survey design using the *survey* package in R. All statistical analyses were performed exploratively in R version 4.0.3.

## Results

### Participant characteristics

A subset of 1912 individuals with the periodontal classification and a whole-body dual X-ray absorptiometry examination from the 2013 to 2014 main cohort were included in the analysis. The mean age of the study cohort was 43.4 (± 8.4) years and 49.4% of the participants were male. Detailed participant characteristics are presented in Table 1. Low handgrip strength and low skeletal muscle mass index were not observed in the study cohort; thus, a classification of sarcopenia was omitted. The SMMI and cHGS were included as continuous variables in the analysis. Overall, 612 (32%) of the participants were determined to have periodontitis, of whom 513 (26.8%) had non-severe (mild or moderate) and 99 (5.2%) had severe periodontitis.

**Table 1:** Participant characteristics stratified by periodontal status

	No periodontitis (*n* = 1300)	Non-severe periodontitis (*n* = 513)	Severe periodontitis (*n* = 99)	*p* Value
Age (in years)	42.69 (± 8.22)	44.20 (± 8.51)	48.13 (± 7.99)	< 0.001
Sex at birth				< 0.001
Female	714 (73.8%)	220 (22.7%)	34 (3.5%)	
Male	586 (62.1%)	293 (31.0%)	65 (6.9%)	
Education level				< 0.001
Low	152 (11.7%)	151 (29.4%)	31 (31.3%)	
Medium	636 (48.9%)	293 (57.1%)	58 (58.6%)	
High	512 (39.4%)	69 (13.5%)	10 (10.1%)	
Type II diabetes mellitus				< 0.001
No diabetes	1011 (77.8%)	357 (69.6%)	60 (60.6%)	
Pre-diabetes	172 (13.2%)	92 (17.9%)	25 (25.3%)	
Diabetes	89 (6.9%)	53 (10.3%)	12 (12.1%)	
Missing	28 (2.2%)	11 (2.1%)	2 (2.0%)	
Body mass index (kg/m^2^)				0.3
≤ 24.9	377 (29.0%)	131 (25.5%)	21 (21.2%)	
25–29.9	438 (33.7%)	178 (34.7%)	33 (33.3%)	
≥ 30	483 (37.2%)	203 (39.6%)	44 (44.4%)	
Missing	2 (0.2%)	1 (0.2%)	1 (1.0%)	
Skeletal muscle mass index				< 0.001
Mean (SD)	18.09 (± 3.35)	18.70 (± 3.30)	19.22 (± 2.94)	< 0.001
Missing	1 (0.1%)	1 (0.2%)	1 (1.0%)	
Combined handgrip strength (kg)				0.4
Mean (SD)	76.22 (± 21.71)	77.26 (± 21.68)	78.59 (± 20.75)	
Missing	52 (4%)	28 (5.5%)	4 (4.0%)	
Bone mineral density (g/cm^2^)				0.6
Mean (SD)	1.12 (± 0.11)	1.11 (± 0.12)	1.12 (± 0.11)	
Missing	12 (0.9%)	4 (0.8%)	0 (0%)	
Total energy intake (kcal/day)				0.1
Mean (SD)	201.66 (± 983.26)	2310.81 (± 1108.61)	2353.13 (± 1123.12)	
Missing	60 (4.6%)	38 (7.4%)	9 (9.1%)	
Total protein intake (g/day)				0.1
Mean (SD)	86.67 (± 43.79)	91.55 (± 48.48)	85.53 (± 41.90)	
Missing	60 (4.6%)	38 (7.4%)	9 (9.1%)	
Vitamin D2 and D3 (nmol/L)				< 0.001
Mean (SD)	62.0 (47.0–77.2)	54.6 (39.0–69.9)	53.6 (37.4–67.6)	

All continuous variables are reported with mean and standard deviation, $\overline{x}$ (± SD), while categorical variables are reported in percentages, no (%)

### Results of unadjusted and fully adjusted multiple linear regression analyses of association between periodontitis and SMMI or cHGS

In unadjusted regression models, non-severe or severe periodontitis were not associated with SMMI (*β* = 1.01, 95% CI 0.50; 1.52 and *β* = 1.42, 95% CI 0.59; 2.25, respectively) but with cHGS by tendency (*β* = 1.83, 95% CI − 0.38; 4.05 and *β* = 2.03, 95% CI − 4.64; 8.69 and, respectively). The results of the unadjusted analyses are presented in Table 2.

**Table 2:** Unadjusted results of linear regression analyses of association between periodontitis and SMMI or cHGS

Predictors	SMMI			cHGS		
	Estimates	95% CI	*p* value	Estimates	95% CI	*p* Value
Intercept	18.06	17.80–18.33	** < 0.001**	77.56	76.02–79.10	** < 0.001**
Non-severe periodontitis	1.01	0.50–1.52	**0.001**	1.83	− 0.38 to 4.05	0.098
Severe periodontitis	1.42	0.59–2.25	**0.003**	2.03	− 4.64 to 8.69	0.523

*Reference group* no periodontitis, *SMMI* skeletal muscle mass index, *cHGS* combined handgrip strength

Bold values are significant if *p* < 0.05

After adjusting for age, sex, education, BMI, BMD, diabetic status, education, total energy intake, total protein intake, and serum vitamin D2 + D3, periodontitis showed relevant associations with cHGS (*β*_non-severe_ = − 2.81, 95% CI − 4.7; − 1.15 and *β*_severe_ = − 2.73, 95% CI − 6.31; 0.83 with severe periodontitis). The fully adjusted regression analysis of the association of SMMI with periodontitis resulted in *β*_non-severe_ = 0.07, 95% CI − 0.26; 0.40 and *β*_severe_ = 0.22, 95% CI − 0.34; 0.78. The detailed results of the adjusted regression analyses of combined handgrip strength and skeletal muscle mass index are presented in Table 3. Further predictors and coefficients of the models are presented in the supplementary tables (Supplementary Tables 1 and 2).

**Table 3:** Fully adjusted results of linear regression analyses of association between periodontitis and SMMI or cHGS

Predictors	SMMI			cHGS		
	Estimates	95% CI	*p* value	Estimates	95% CI	*p* value
Intercept	4.30	1.85 – 6.76	**0.017**	62.32	51.29 – 73.35	** < 0.001**
Non-severe periodontitis	0.07	− 0.26 to 0.40	0.451	− 2.81	− 4.47 to − 1.15	**0.005**
Severe periodontitis	0.22	− 0.34 to 0.78	0.231	− 2.73	− 6.31 to 0.83	**0.154**

*Reference group* no periodontitis, *SMMI* skeletal muscle mass index, *cHGS* combined handgrip strength

Bold values are significant if *p* < 0.05

## Discussion

In the present study, we investigated the association between periodontitis and sarcopenia indicators (cHGS and SMMI), without using tooth loss as a proxy and taking common risk factors and demographic characteristics into consideration. The results indicated that periodontitis was associated with cHGS but not with SMMI in adults below 60 years of age, after adjusting for covariates that were associates of muscle function and muscle metabolism as well as common risk factors of sarcopenia and periodontitis, such as diabetes mellitus and BMI.

A possible explanation for the association between the cHGS and periodontitis could be the reduced oral function and worsened nutrition as a result of tooth loss. Although the reasons of tooth loss are often unspecified in most studies in the literature, periodontitis is a leading cause of tooth loss in adults [34]. Adults with tooth loss are at greater risk of malnutrition [35], an important risk factor for sarcopenia and frailty [36]. Tooth mobility in periodontitis patients has been associated with a reduced bite force [37]. The reduced mechanoreceptive activity in the periodontal ligament can result in reduced periodontal support, which impairs the regulation of masticatory forces [38]. Function is an important predictor of muscle strength and skeletal and masticatory muscle endurance capacity [39]. Thus, reduced masticatory function due to periodontitis can contribute to oral hypofunction and sarcopenia [40]. A recent study reported that oral hypofunction was significantly associated with frailty [41]. In a study of a nationally representative cohort, participants with fewer than nine teeth showed significant associations with lower handgrip strength in Korean men (OR: 1.39, 95% CI 1.03; 1.88, mean age 72.9 ± 0.1) after adjusting for covariates [42]. In a study of 2,089 adult men and women between the ages of 30 and 90 in northern Germany, higher clinical attachment loss was associated with lower handgrip strength [43]. In sarcopenic Korean adults, periodontitis prevalence was significantly higher compared to non-sarcopenic adults (30.3% vs. 18.3% in males, 45.9% vs. 17.4% in females, *p* < 0.001) [44]. The same study reported that having fewer than 20 teeth was associated with a higher incidence of sarcopenia in adults aged 65 years and older (OR 1.92, 95% CI 1.49; 2.66 in men and OR 2.63, 95% CI 2.25; 3.64 in women) [44].

Not only epidemiological studies but also in vivo experiments suggest that periodontitis may possibly be associated with muscle strength and mass. In an experimental ligature-induced periodontitis model, reduced strength, reduced number of capillaries, and increased number of inflammatory cells and fibroblasts in skeletal striated muscles were detected in immobilized Wistar rats with periodontitis compared to those without periodontitis, suggesting that periodontitis could possibly contribute to muscle atrophy [45]. In mice, the injection of the lipopolysaccharide of *Porphyromonas (P.) gingivalis*, a major periodontal pathogen, into the masseter and tibialis anterior muscles decreased muscle weight and increased fibrotic area and myocyte apoptosis eightfold in the masseter muscle, mediated in part by the activation of the toll-like receptor 4 (TLR4)/extracellular signal-regulated kinase (ERK) pathway [46]. Oral administration of *P. gingivalis* also altered glucose uptake signaling and gene expression in soleus muscle tissue in mice, resulting in increased mRNA expression of tumor necrosis factor alpha (*Tnfa),* interleukin-6 *(Il6)*, C-C motif chemokine 2 (*Ccl2*), and myogenin (*Myog*) compared to administration of saline [47]. Approximately, 79% of periodontitis patients may harbor *P. gingivalis* [48], a highly virulent pathogen that can also invade peripheral tissues, e.g., atherosclerotic plaques [49] and synovial fluids [50], and contribute to the pathogenesis of cardiovascular and rheumatic diseases.

This study has several limitations. First, there were relatively fewer participants in the severe periodontitis group compared to non-severe periodontitis group, which may have influenced the results. Second, a causal relationship between periodontitis and cHGS or SMMI cannot be inferred from this cross-sectional analysis. In addition, the sub-cohort included in the present study lacked participants with low SSMI or low cHGS, because it included only dentate U.S. adults below 60 years of age, who underwent both a clinical periodontal examination and a whole-body DXA scan. Therefore, our results are only representative for the studied sub-cohort (*n* = 1912), but not for the entire NHANES 2013–2014 cohort (n = 10,683). This affects the generalization of the results and introduces selection bias. In older ages, the association between muscle mass and muscle strength becomes more prominent than at younger ages; hence, this may result in a disparity in the association estimates of SMMI and cHGS with periodontitis. Finally, a number of independent factors influence muscle strength and mass, such as physical activity, genetics and hormones, which could not be accounted for in the present study. However, our results align with previous studies conducted in different populations and age groups that investigated the association between periodontitis proxies, i.e., tooth loss, and sarcopenia indicators, i.e., handgrip strength, despite these limitations.

## Conclusions

The findings of the current study indicate that periodontitis and sarcopenia indicators may be interrelated. Further observational studies are needed to clarify the nature and direction of an association. Future studies can contribute to findings that support the screening, prevention and clinical management of sarcopenia and periodontitis, and emphasize the interdisciplinary and complementary approach between the disciplines of geriatric medicine and periodontology.

## Supplementary Information

Below is the link to the electronic supplementary material.

Supplementary file1 (DOCX 32 KB)

## Data Availability

The data used in this study is publicly available under the following link: https://www.cdc.gov/nchs/NHANES/.
