# Supplementary material for: Association of periodontitis with handgrip strength and skeletal muscle mass in middle-aged US adults from NHANES 2013–2014
Source: Aging Clin Exp Res. 2023 Jun 30;35(9):1909–16. doi: 10.1007/s40520-023-02471-2 (PMC10460310; doi:10.1007/s40520-023-02471-2)
Supplement: Supplementary file 1 — Supplementary file1 (DOCX 32 KB) [file 40520_2023_2471_MOESM1_ESM.docx]

**Supplementary Material**

**Title:**

Association of periodontitis with handgrip strength and skeletal muscle mass in middle-aged U.S. adults from NHANES 2013–2014

**Authors:**

Kübra Bunte^1*^, Christian Wiessner^1^, Gülistan Bahat^2^, Tugba Erdogan^2^, Alfonso J Cruz-Jentoft^3^, Antonia Zapf^1^

**Affiliations:**

^1^The Center for Experimental Medicine, Medical Biometry and Epidemiology, University Medical Center Hamburg-Eppendorf, Hamburg, Germany

^2^Department of Internal Medicine, Istanbul Medical Faculty, Division of Geriatrics, Istanbul University, Istanbul, Turkey

^3^Servicio de Geriatría, Hospital Universitario Ramón y Cajal (IRYCIS), Madrid, Spain

**Supplementary Table 1.** Results of fully adjusted linear regression model of association between combined handgrip strength and periodontitis

|  | **cHGS** | | |
| --- | --- | --- | --- |
| ***Predictors*** | ***Estimates*** | ***CI*** | ***p*** |
| Intercept | 62.32 | 51.29 – 73.35 | **<0.001** |
| Periodontitis [Non-severe periodontitis] | -2.81 | -4.47 – -1.15 | **0.005** |
| Periodontitis [Severe periodontitis] | -2.73 | -6.31 – 0.83 | **0.154** |
| Sex at birth [Women] | -37.34 | -43.42 – -31.27 | **<0.001** |
| Age (in years) | -0.32 | -0.46 – -0.19 | **<0.001** |
| BMD, g/cm^2^ | 30.04 | 22.96 – 37.11 | **<0.001** |
| BMI, kg/m2 | 0.39 | 0.28 – 0.49 | **<0.001** |
| Type II Diabetes status [Diabetes] | -1.57 | -4.59 – 1.41 | 0.316 |
| Type II Diabetes status [Prediabetes] | -0.04 | -2.12 – 2.02 | 0.968 |
| Education level [Low] | -0.22 | -2.65 –2.21 | 0.861 |
| Education level [Medium] | 0.53 | -0.57 – 1.64 | 0.361 |
| Age (in years) * Sex at birth [Women] | 0.08 | -0.05 – 0.21 | **0.265** |
| Total energy intake (kcal/day) | 0.00 | -0.00 – 0.00 | 0.577 |
| Total protein intake (g/day) | -0.01 | -0.03 – 0.02 | 0.564 |
| Vit D2 and D3 (nmol/l) | 0.04 | 0.01 – 0.06 | **0.022** |
| Number of observations | 1676 | | |
| R^2^ | 0.725 | | |
| cHGS: combined handgrip strength, CI: 95% confidence interval, SMMI: skeletal muscle mass index, BMD: bone mineral density, BMI: body-mass index, Vit D2 and D3: total serum vitamin D2 and D3. Reference categories: No periodontitis, Male, No diabetes, High education. | | | |

**Supplementary Table 2.** Results of fully adjusted linear regression model of association between skeletal muscle mass index and periodontitis

|  | **SMMI** | | |
| --- | --- | --- | --- |
| ***Predictors*** | ***Estimates*** | ***CI*** | ***p*** |
| Intercept | 4.30 | 1.85 – 6.76 | **0.017** |
| Periodontitis [Non-severe periodontitis] | 0.07 | -0.26 – 0.40 | 0.451 |
| Periodontitis [Severe periodontitis] | 0.22 | -0.34 – 0.78 | 0.231 |
| Sex at birth [Women] | -3.02 | -3.34 – -2.69 | **0.001** |
| Age (in years) | -0.01 | -0.03 – 0.00 | 0.072 |
| BMD, g/cm^2^ | 3.14 | 1.91 – 4.36 | **0.008** |
| BMI, kg/m2 | 0.42 | 0.38 – 0.45 | **<0.001** |
| Type II Diabetes status [Diabetes] | 0.51 | -0.11 – 1.13 | 0.070 |
| Type II Diabetes status [Prediabetes] | 0.31 | -0.12 – 0.74 | 0.090 |
| Education level [Low] | 0.04 | -0.40 – 0.49 | 0.708 |
| Education level [Medium] | -0.08 | -0.45 – 0.28 | 0.429 |
| Total energy intake (kcal/day) | 0.00 | -0.00 – 0.00 | 0.319 |
| Total protein intake (g/day) | 0.00 | -0.00 – 0.01 | 0.530 |
| Vit D2 and D3 (nmol/l) | 0.00 | -0.00 – 0.01 | 0.168 |
| Number of observations | 1742 | | |
| R^2^ | 0.710 | | |
| CI: 95% confidence interval, SMMI: skeletal muscle mass index, BMD: bone mineral density, BMI: body-mass index, Vit D2 and D3: total serum vitamin D2 and D3. Reference categories: No periodontitis, Male, No diabetes, High education. | | | |

**R Code**

library(SASxport)

library(tidyverse)

library(survey)

library(broom)

library(gt)

library(labelled)

library(gtsummary)

library(Gmisc)

library(sjPlot)

library(sjmisc)

library(sjlabelled)

lookup.xport("/Users/kubrabunte/Desktop/NHANES/oral.xpt")

lookup.xport("/Users/kubrabunte/Desktop/NHANES/dx.xpt")

lookup.xport("/Users/kubrabunte/Desktop/NHANES/musclegripdata.xpt")

lookup.xport("/Users/kubrabunte/Desktop/NHANES/bodymeasuresdata.xpt")

lookup.xport("/Users/kubrabunte/Desktop/NHANES/demographicsdata.xpt")

lookup.xport("/Users/kubrabunte/Desktop/NHANES/glycohemoglobindata.xpt")

lookup.xport("/Users/kubrabunte/Desktop/NHANES/nutrientintakedata.xpt")

lookup.xport("/Users/kubrabunte/Desktop/NHANES/vitddata.xpt")

lookup.xport("/Users/kubrabunte/Desktop/NHANES/cotinine.xpt")

lookup.xport("/Users/kubrabunte/Desktop/NHANES/cbcdata.xpt")

lookup.xport("/Users/kubrabunte/Desktop/NHANES/biochemdata.xpt")

lookup.xport("/Users/kubrabunte/Desktop/NHANES/diabetesquestionnairedata.xpt")

lookup.xport("/Users/kubrabunte/Desktop/NHANES/physicalactivityquestdata.xpt")

lookup.xport("/Users/kubrabunte/Desktop/NHANES/disabilitydata.xpt")

lookup.xport("/Users/kubrabunte/Desktop/NHANES/smokingdata.xpt")

lookup.xport("/Users/kubrabunte/Desktop/NHANES/medicalcondata.xpt")

lookup.xport("/Users/kubrabunte/Desktop/NHANES/bpdata.xpt")

lookup.xport("/Users/kubrabunte/Desktop/NHANES/prescriptiondata.xpt")

oraldata <- read.xport ("/Users/kubrabunte/Desktop/NHANES/oral.xpt")

dxdata <- read.xport("/Users/kubrabunte/Desktop/NHANES/dx.xpt")

gripdata <- read.xport("/Users/kubrabunte/Desktop/NHANES/musclegripdata.xpt")

bodydata <- read.xport("/Users/kubrabunte/Desktop/NHANES/bodymeasuresdata.xpt")

demogdata <- read.xport("/Users/kubrabunte/Desktop/NHANES/demographicsdata.xpt")

hbA1cdata <- read.xport("/Users/kubrabunte/Desktop/NHANES/glycohemoglobindata.xpt")

nutrientdata <- read.xport("/Users/kubrabunte/Desktop/NHANES/nutrientintakedata.xpt")

vitddata <- read.xport("/Users/kubrabunte/Desktop/NHANES/vitddata.xpt")

nicotinedata <- read.xport("/Users/kubrabunte/Desktop/NHANES/cotinine.xpt")

cbcdata <- read.xport("/Users/kubrabunte/Desktop/NHANES/cbcdata.xpt")

biochemdata <- read.xport("/Users/kubrabunte/Desktop/NHANES/biochemdata.xpt")

diabquestdata <-read.xport("/Users/kubrabunte/Desktop/NHANES/diabetesquestionnairedata.xpt")

physicalactquestdata <- read.xport("/Users/kubrabunte/Desktop/NHANES/physicalactivityquestdata.xpt")

disabilitydata <- read.xport("/Users/kubrabunte/Desktop/NHANES/disabilitydata.xpt")

smokingdata <- read.xport("/Users/kubrabunte/Desktop/NHANES/smokingdata.xpt")

medicalcondata <- read.xport("/Users/kubrabunte/Desktop/NHANES/medicalcondata.xpt")

bpdata <- read.xport("/Users/kubrabunte/Desktop/NHANES/bpdata.xpt")

prescrdata <- read.xport("/Users/kubrabunte/Desktop/NHANES/prescriptiondata.xpt")

mydata <- merge(demogdata, oraldata, by= "SEQN",all = TRUE)

mydata <- merge(mydata, gripdata, by="SEQN",all = TRUE)

mydata <- merge(mydata, bodydata, by="SEQN",all = TRUE)

mydata <- merge(mydata, dxdata, by="SEQN", all.x=TRUE, all.y=TRUE)

mydata <- merge(mydata, hbA1cdata, by="SEQN",all = TRUE)

mydata <- merge(mydata, nutrientdata, by="SEQN",all = TRUE)

mydata <- merge(mydata, vitddata, by="SEQN",all = TRUE)

mydata <- merge(mydata, nicotinedata, by="SEQN",all = TRUE)

mydata <- merge(mydata, cbcdata, by="SEQN",all = TRUE)

mydata <- merge(mydata, biochemdata, by="SEQN",all = TRUE)

mydata <- merge(mydata, diabquestdata, by="SEQN",all = TRUE)

mydata <- merge(mydata, physicalactquestdata, by="SEQN",all = TRUE)

mydata <- merge(mydata, disabilitydata, by="SEQN",all = TRUE)

mydata <- merge(mydata, smokingdata, by="SEQN",all = TRUE)

mydata <- merge(mydata, medicalcondata, by="SEQN",all = TRUE)

mydata <- merge(mydata, bpdata, by="SEQN",all = TRUE)

# Removing participants with missing data from periodontal examinations

mydata <- mydata[!mydata$OHDPDSTS == 2, ]

mydata <- mydata[!mydata$OHDPDSTS == 3, ]

mydata <- mydata[!is.na(mydata$OHDPDSTS),]

# Removing participants with missing dual x-ray absorptiometry data

mydata<- mydata[mydata$DXAEXSTS ==1, ]

mydata <- mydata[!is.na(mydata$DXDTOLE),]

##### Application of Exclusion Criteria #####

# 1.Exclude medical conditions

# MCQ160B(heart failure)

mydata <- mydata[mydata$MCQ160B != 1,]

# MCQ160C(coronary heart disease),

mydata <- mydata[mydata$MCQ160C != 1,]

# MCQ160D (angina pectoris),

mydata <- mydata[mydata$MCQ160D != 1,]

# MCQ160E (heart attack),

mydata <- mydata[mydata$MCQ160E != 1,]

# MCQ160F (stroke),

mydata <- mydata[mydata$MCQ160F != 1,]

# MCQ220 (cancer/malignancy)

mydata <- mydata[mydata$MCQ220 != 1,]

# 2. Exclude corticosteroid use:

#d00254-hydrocortisone, d00206-dexamethasone,

#d00608-fluodrocortisone, d00609-cortisone

drugs <- c("d00254", "d00206", "d00608", "d00609")

mydata <- mydata[!(mydata %in% drugs), ]

##### Periodontal Classification #####

# 1. Buildung up vectors for the probes to check (names of the teeth according to American system)

n_mesio <- c(2:15, 18:31)

n_distal <- c(3:14, 19:30)

neighbor_mesio <- c(replicate(7, 1), replicate(7, -1), replicate(7, 1), replicate(7, -1))

neighbor_distal <- c(replicate(6, -1), replicate(6, 1), replicate(6, -1), replicate(6, 1))

# 2. Column names for interproximal AL probes

al_cn_self <- c(

sprintf("OHX%02dLAD", n_distal),

sprintf("OHX%02dLAP", n_distal),

sprintf("OHX%02dLAS", n_mesio),

sprintf("OHX%02dLAA", n_mesio)

)

# 3. Column names for neighboring AL probes to check if they exist

al_cn_test <- c(

sprintf("OHX%02dLAS", n_distal + neighbor_distal),

sprintf("OHX%02dLAA", n_distal + neighbor_distal),

sprintf("OHX%02dLAD", n_mesio + neighbor_mesio),

sprintf("OHX%02dLAP", n_mesio + neighbor_mesio)

)

# 4. Column names for interproximal PD probes

pd_cn_self <- c(

sprintf("OHX%02dPCD", n_distal),

sprintf("OHX%02dPCP", n_distal),

sprintf("OHX%02dPCS", n_mesio),

sprintf("OHX%02dPCA", n_mesio)

)

# 5. Column names for neighboring PD probes to check if they exist

pd_cn_test <- c(

sprintf("OHX%02dPCS", n_distal + neighbor_distal),

sprintf("OHX%02dPCA", n_distal + neighbor_distal),

sprintf("OHX%02dPCD", n_mesio + neighbor_mesio),

sprintf("OHX%02dPCP", n_mesio + neighbor_mesio)

)

all_pds <- c(

sprintf("OHX%02dPCD", n_mesio),

sprintf("OHX%02dPCP", n_mesio),

sprintf("OHX%02dPCM", n_mesio),

sprintf("OHX%02dPCL", n_mesio),

sprintf("OHX%02dPCS", n_mesio),

sprintf("OHX%02dPCA", n_mesio)

)

all_als <- c(

sprintf("OHX%02dLAD", n_mesio),

sprintf("OHX%02dLAP", n_mesio),

sprintf("OHX%02dLAM", n_mesio),

sprintf("OHX%02dLAL", n_mesio),

sprintf("OHX%02dLAS", n_mesio),

sprintf("OHX%02dLAA", n_mesio)

)

# 6. Function to execute for each row to build up a list of columns of interproximal columns

getInterproximalColNames <- function(x, cn_self, cn_test) {

v <- c()

for (i in 1:length(cn_self)) {

v <- c(v, ifelse(x[cn_self[i]] < 99 & x[cn_test[i]] < 99, cn_self[i], ""))

}

v <- v[!v %in% ""]

c(v, use.names = FALSE)

}

# 7. Executing the function and remove patients that have no interproximal AL probes

mydata$interp_al_names <- unname(apply(mydata, 1, getInterproximalColNames, al_cn_self, al_cn_test))

mydata <- mydata[lengths(mydata$interp_al_names) > 0, ]

mydata$interp_pd_names <- unname(apply(mydata, 1, getInterproximalColNames, pd_cn_self, pd_cn_test))

mydata <- mydata[lengths(mydata$interp_pd_names) > 0, ]

# 8. Function to calculate how many columns have an value equal or greater than a certain value

calcNumGt <- function(x, col.name, min_value) {

sum(unlist(x[x[[col.name]]]) >= min_value)

}

calcNumTeethGt <- function(x, col.name, min_value) {

tooth <- as.numeric(substr(x[[col.name]], 4, 5))

d <- data.frame(probe=x[[col.name]], tooth=tooth, pd=unlist(x[x[[col.name]]]))

max_pd_per_tooth <- aggregate(d$pd, list(d$tooth), max)

sum(max_pd_per_tooth[2] >= min_value)

}

calcMaxPd <- function(x, col.names) {

pds <- unlist(x[col.names])

max(pds[pds != 99])

}

calcMeanValue <- function(x, col.names) {

values <- unlist(x[col.names])

mean(values[values != 99])

}

# 9. Apply function for 3mm, 4mm and 6mm AL

mydata$num_al_3mm <- apply(mydata, 1, calcNumGt, "interp_al_names", 3)

mydata$num_teeth_al_4mm <- apply(mydata, 1, calcNumTeethGt, "interp_al_names", 4)

mydata$num_teeth_al_6mm <- apply(mydata, 1, calcNumTeethGt, "interp_al_names", 6)

# 10. Apply function for 4mm and 5mm AL

mydata$num_teeth_pd_4mm <- apply(mydata, 1, calcNumTeethGt, "interp_pd_names", 4)

mydata$num_teeth_pd_5mm <- apply(mydata, 1, calcNumTeethGt, "interp_pd_names", 5)

# 11. Maximum PD for this patient

mydata$max_pd <- apply(mydata, 1, calcMaxPd, all_pds)

# 12. Classify periodontitis in 4 categories

classifyPerio <- function(x) {

if (x$num_teeth_al_6mm >= 2 & x$num_teeth_pd_5mm >= 1) {

return("Severe")

} else if (x$num_teeth_al_4mm >= 2 | x$num_teeth_pd_5mm >= 2) {

return("Moderate")

} else if (x$num_al_3mm >= 2 & (x$num_teeth_pd_4mm >= 2 | x$max_pd >= 5)) {

return("Mild")

} else {

return("No")

}

}

###### Periodontal Classification after applying all exclusion criteria #######

mydata$periocat4 <- apply(mydata, 1, classifyPerio)

mydata$perioStatus <- mydata$periocat4 != "No"

# Classification as no, Non-Severe and Severe periodontitis

mydata$periocat3 <- ifelse(mydata$periocat4 == "Mild" | mydata$periocat4 == "Moderate", "Non-Severe", mydata$periocat4)

mydata$periocat3 <- ifelse(mydata$periocat4 == "Severe", "Severe", mydata$periocat3)

mydata$periocat3 <- ifelse(mydata$periocat4 == "No", "No", mydata$periocat3)

mydata$periocat3 <- factor(mydata$periocat3)

mydata$periocat3 <- relevel(mydata$periocat3, ref = "No")

# Skeletal muscle mass index

summary(mydata$DXDTOLE, exclude=NULL)

summary(mydata$BMXHT, exclude=NULL)

mydata$heightinmeters <- as.numeric(mydata$BMXHT)/100

summary(mydata$heightinmeters)

mydata$musclemassinkg <- mydata$DXDTOLE/1000

summary(mydata$musclemassinkg)

mydata$HASMM <- as.numeric(mydata$musclemassinkg)/(as.numeric(mydata$heightinmeters)^2)

summary(mydata$HASMM, exclude=NULL)

#Sex at birth

mydata$RIAGENDR <- as.factor(mydata$RIAGENDR)

levels(mydata$RIAGENDR)[levels(mydata$RIAGENDR)==1] <- "Men"

levels(mydata$RIAGENDR)[levels(mydata$RIAGENDR)==2] <- "Women"

# Education

mydata$education <- ifelse(mydata$DMDEDUC2 %in% c("1","2"), "low", mydata$DMDEDUC2)

mydata$education <- ifelse(mydata$DMDEDUC2 %in% c("3","4"), "medium", mydata$education)

mydata$education <- ifelse(mydata$DMDEDUC2 == 5, "high", mydata$education)

#BMI

mydata$BMIcat <- ifelse(mydata$BMXBMI <= 24.9, "<=24.9",mydata$BMXBMI)

mydata$BMIcat <- ifelse(mydata$BMXBMI >= 25 & mydata$BMXBMI <= 29.9, "25-29.9", mydata$BMIcat)

mydata$BMIcat <- ifelse(mydata$BMXBMI >= 30, "30>=", mydata$BMIcat)

#HbA1c

table(mydata$LBXGH)

mydata$diabcat <- ifelse(mydata$LBXGH >= 6.5 , "Diabetes", mydata$LBXGH)

mydata$diabcat <- ifelse(mydata$LBXGH < 6.5 & mydata$LBXGH > 5.7 , "Prediabetes", mydata$diabcat)

mydata$diabcat <- ifelse(mydata$LBXGH <= 5.7 , "Non-diabetes", mydata$diabcat)

mydata$diabcat <- as.factor(mydata$diabcat)

mydata$diabcat <- relevel(mydata$diabcat, ref = "Non-diabetes")

table(mydata$diabcat)

# 1. Participant characteristics

getTable1Stats <- function(x, digits = 2, ...){

getDescriptionStatsBy (x = x,

by = mydata$periocat3,

digits = digits,

continuous_fn = describeMean,

prop_fn = describeProp,

header_count = TRUE,

statistics = TRUE,

missing_value = "-",

...)

}

table1 <- list()

table1[["Age"]] <-

getTable1Stats(mydata$RIDAGEYR)

table1[["Sex at birth"]] <-

getTable1Stats(mydata$RIAGENDR)

table1[["Education"]] <-

getTable1Stats(mydata$education)

table1[["Diabetes Mellitus"]] <-

getTable1Stats(mydata$diabcat)

table1[["BMI"]] <-

getTable1Stats(mydata$BMIcat)

table1[["SMMI"]] <-

getTable1Stats(mydata$HASMM)

table1[["Handgrip strength"]] <-

getTable1Stats(mydata$MGDCGSZ)

table1[["Bone mineral Density"]] <-

getTable1Stats(mydata$DXDTOBMD)

table1[["Total Daily Energy Intake"]] <-

getTable1Stats(mydata$DR1TKCAL)

table1[["Total Daily Protein Intake"]] <-

getTable1Stats(mydata$DR1TPROT)

table1[["Vitamin D2 and D3"]] <-

getTable1Stats(mydata$LBXVIDMS)

mergeDesc(table1,

getTable1Stats(mydata$periocat3)) %>%

htmlTable(caption = "Participant characteristics stratified by periodontitis",

tfoot = c("All continuous variables are reported with mean and standard deviation, x̄ (± SD), while categorical variables are reported in percentages, no (%) "),

ctable = TRUE)

table1 <- data.frame(table1)

# Survey design

mydata <- subset(mydata , !is.na(WTMEC2YR))

mydata <- subset(mydata , !is.na(SDMVPSU))

nhanesDesign <- svydesign (id = ~SDMVPSU,

strata = ~SDMVSTRA,

weights = ~WTMEC2YR,

nest = TRUE,

data = mydata)

nhanesDesign

ageDesign <- subset(nhanesDesign, RIDAGEYR > 29 &

RIDAGEYR < 60)

# Unadjusted models

smmi <- svyglm (HASMM ~ periocat3,

family = gaussian(),

data = nhanesDesign,

design = ageDesign

)

summary(smmi)

plot(smmi)

tab_model(smmi)

hgs <- svyglm (MGDCGSZ ~ periocat3,

family = gaussian(),

data = nhanesDesign,

design = ageDesign

)

summary(hgs)

plot(hgs)

tab_model(hgs)

# Fully adjusted models

adjustedsmmi <- svyglm (HASMM ~ periocat3

+ RIAGENDR + RIDAGEYR + DXDTOBMD + BMXBMI +

diabcat + education + DR1TKCAL + DR1TPROT +

LBXVIDMS,

family = gaussian(),

data = nhanesDesign,

design = ageDesign

)

summary(adjustedsmmi)

plot(adjustedsmmi)

tab_model(adjustedsmmi)

adjustedhgs <- svyglm (HASMM ~ periocat3

+ RIAGENDR + RIDAGEYR + DXDTOBMD + BMXBMI +

diabcat + education + DR1TKCAL + DR1TPROT +

LBXVIDMS,

family = gaussian(),

data = nhanesDesign,

design = ageDesign

)

summary(adjustedhgs)

plot(adjustedhgs)

tab_model(adjustedhgs)
